# Supplementary material for: Addressing Outcome Reporting Bias in Meta‐Analysis: A Selection Model Perspective
Source: Stat Med. 2025 Dec 15;44(28-30):e70238. doi: 10.1002/sim.70238 (PMC12704405; doi:10.1002/sim.70238)

Addressing Outcome Reporting Bias in Meta-analysis: A Selection Model Perspective

Supplementary Figures and Results

Alessandra Gaia Saracini[1] and Leonhard Held[2]

In the manuscript's Results section, we focus primarily on the bias in estimating the treatment effect μ. This supplementary document presents additional performance metrics from the simulation study, including mean squared error (MSE), coverage, power, and empirical standard error (ESE).

We also include results from a simulation scenario where missingness follows a missing completely at random (MCAR) mechanism. Here, we apply both naive estimation and the ORB-correction methods developed for the MNAR setting. This provides a benchmark for the primary analysis and illustrates the effect of correcting for ORB when no such bias is present. These findings highlight the value of using ORB-adjustment as part of a sensitivity analysis when the true missing data mechanism is uncertain.

1 Additional Measures of Primary Simulation (MNAR)

The MSE of the naive estimate of treatment effect is substantially reduced in high heterogeneity settings, for all ORB-adjusted estimates, as can be seen from Figure 1, for the ORB DGM with $\gamma = 1.5$, and from Figure 2, for the ORB DGM with $\gamma = 0.5$. Similar to the results of the bias measures, we note better performance of the ORB-adjusted methods for $K = 15, 30$, with more limited improvements for the small meta-analysis size $K = 5$.

Coverage for the ORB DGM with $\gamma = 0.5$ is shown in Figure 3. As for the $\gamma = 1.5$ setting, shown in the main manuscript, coverage can be substantially low with naive estimation. There are no substantial differences between the various ORB-adjusted estimates obtained with the different selection functions, including the correct model specification one. Overall, the coverage is very high when adjusting for ORB. We observe a dip in the coverage of the ORB-adjusted estimates around $\mu = 0.4$, which can be explained by the fact that, for smaller μ, with various unreported study outcomes, we expect higher widths of the CI and thus higher coverage, while for larger μ, there are fewer unreported studies, so overall less bias and thus higher coverage.

Figures 4 and 5 show the power of the various estimates. We observe that with naive estimation, for both ORB DGM processes $\gamma = 1.5$ and $\gamma = 0.5$, the power is severely inflated. The power of the naive estimate is particularly high for elevated heterogeneity settings and for large meta-analysis study sizes. For the $\gamma = 1.5$ setting, we observe that the correct DGM selection function results in the lowest power among the various ORB-adjusted estimates, with other ORB-adjusted estimates providing in-between results. For the ORB DGM with $\gamma = 0.5$, the opposite is observed, i.e., the correctly specified selection function w_{DGM} results in highest power, with other ORB-adjusted estimates being lower. This is consistent with the bias findings,

[1]Corresponding author: Alessandra Gaia Saracini
University of Zurich, Epidemiology, Biostatistics and Prevention Institute
ETH Zurich, Department of Mathematics
alessandragaia.saracini@gmail.com
[2]Leonhard Held, Professor
University of Zurich, Epidemiology, Biostatistics and Prevention Institute
leonhard.held@uzh.ch

as most ORB-adjusted estimates were too conservative in the adjustment, shifting the estimate towards the null, with a thus increased likelihood of obtaining ORB-adjusted results which are not significant.

The empirical standard error (ESE) performance measure is shown in Figures 6 and 7. We first note that the ESE of the naive estimate is overall consistent with the calculations done for the expected SE of $\hat{\mu}$ in the simulation study protocol. In this case, the naive estimate has, overall, slightly higher SE, given the presence of unreported study outcomes; furthermore, the naive ESE in some settings decreases slightly as μ increases, given the increasing amount of reported study outcomes. In most settings, there are no substantial differences in the ESE between the various ORB-adjusted estimates and the naive one. Some exceptions which can be noted are that for i) the small meta-analysis study size, i.e., $K = 5$, and ii) the high heterogeneity, e.g., $I^2 = 90$, the various ORB-adjusted estimates all have a similar ESE, which is lower than the naive one. On the other hand, for the same heterogeneity level but $K = 15, 30$, some ORB-adjusted estimates have higher ESE than the naive one, in particular for the ORB DGM setting $\gamma = 0.5$. This could indicate a possible threshold behavior, wherein adding contributions from a few unreported study outcomes can increase precision of the estimate, as we are using additional information which was not known with naive estimation; however, having many unreported outcomes contributing to the likelihood results in less precision due to uncertainty in the information added.

The plot 8 shows the bias in the estimation of the heterogeneity variance τ^2 in the presence of ORB, with a DGM for ORB using the parameter setting $\gamma = 0.5$. We observe that for $K = 15, 30$ heterogeneity tends to be overestimated for all estimation methods, while it is underestimated for $K = 5$ and large μ values. The correctly specified ORB-adjusted model estimates the lowest heterogeneity and is closest to the zero-bias line for $K = 15$ and $K = 30$, but furthest from it for $K = 5$. This confirms the somewhat different behavior which can be observed for small meta-analysis of $K = 5$, thus encouraging caution in this setting.

2 Measures of Secondary Simulation (MCAR)

In our primary simulation study, we assessed the impact of outcome reporting bias (ORB), generated under a missing not at random (MNAR) mechanism, on the estimation of the treatment effect μ. The naive estimator, which assumes missing completely at random (MCAR), showed substantial upward bias. In contrast, ORB-adjustment methods, designed for MNAR settings, substantially reduced this bias and, under correct model specification, nearly eliminated it, except in small-sample scenarios (i.e., $K = 5$). As a benchmark, we simulated data under a MCAR mechanism and applied both the naive and ORB-adjusted estimators using the same parameter values (Figure 9). As expected, the naive estimator was unbiased, while the ORB-adjusted estimators produced modest downward bias, particularly under low heterogeneity. These results suggest that ORB adjustment is effective when ORB is present but reasonably robust when it is not. Therefore, we encourgae the application of ORB-adjustment techniques when ORB is suspected, but underscore the importance of treating ORB adjustment as a sensitivity analysis, given the often unknown nature of the missingness mechanism. Of note, even under MCAR, small samples with near-null true treatment effects led to upward bias in the naive estimator, highlighting the need for caution in interpreting results of our simulations for the $K = 5$ scenario.

Regarding the estimation of heterogeneity (τ^2), illustrated in Figure 10 we found little difference between the naive estimator (correctly assuming MCAR) and the ORB-adjusted estimators (which misspecify the mechanism as MNAR). Across most scenarios, τ^2 was underestimated, likely reflecting the general tendency of maximum likelihood estimation to underestimate heterogeneity. Interestingly, in the primary ORB simulations, τ^2 was also often underestimated, but this bias was largely corrected when the ORB-adjusted model aligned with the true MNAR data-generating mechanism. These findings support the use of ORB-adjusted selection models not only for estimating treatment effects, but also for improving heterogeneity estimation in the presence of outcome reporting bias.

Figure 1: MSE in the estimation of the treatment effect μ for ORB simulated according to DGM function with $\gamma = 1.5$, using different estimation methods, i.e., naive or ORB-adjusted according to the various selection functions indicated in the legend. The MSE is shown for varying meta-analysis study sizes, heterogeneity levels, and increasing true treatment effect on the x-axis of each plot shown.

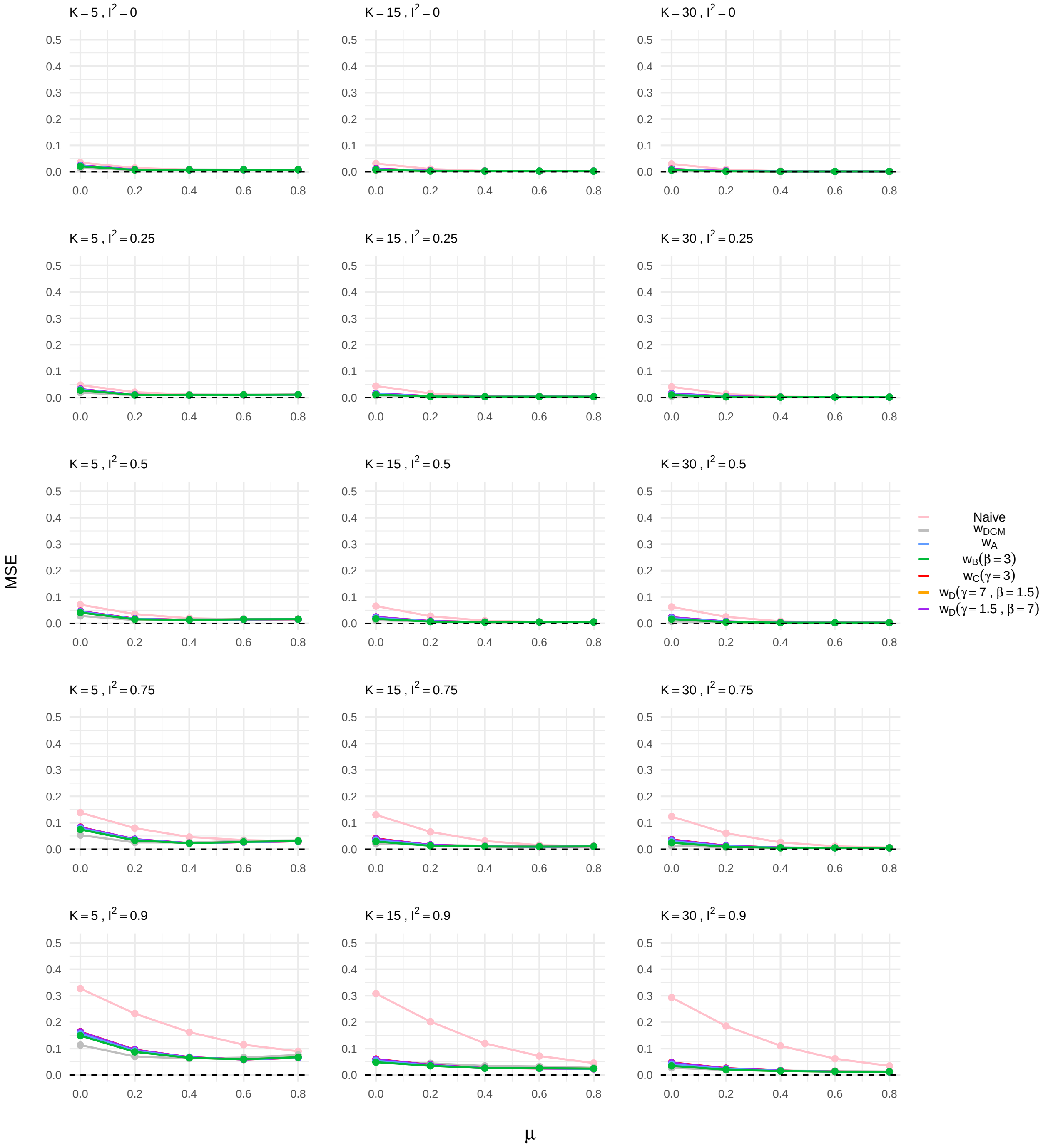

Figure 2: MSE in the estimation of the treatment effect μ for ORB simulated according to DGM function with $\gamma = 0.5$, using different estimation methods, i.e., naive or ORB-adjusted according to the various selection functions indicated in the legend. The MSE is shown for varying meta-analysis study sizes, heterogeneity levels, and increasing true treatment effect on the x-axis of each plot shown.

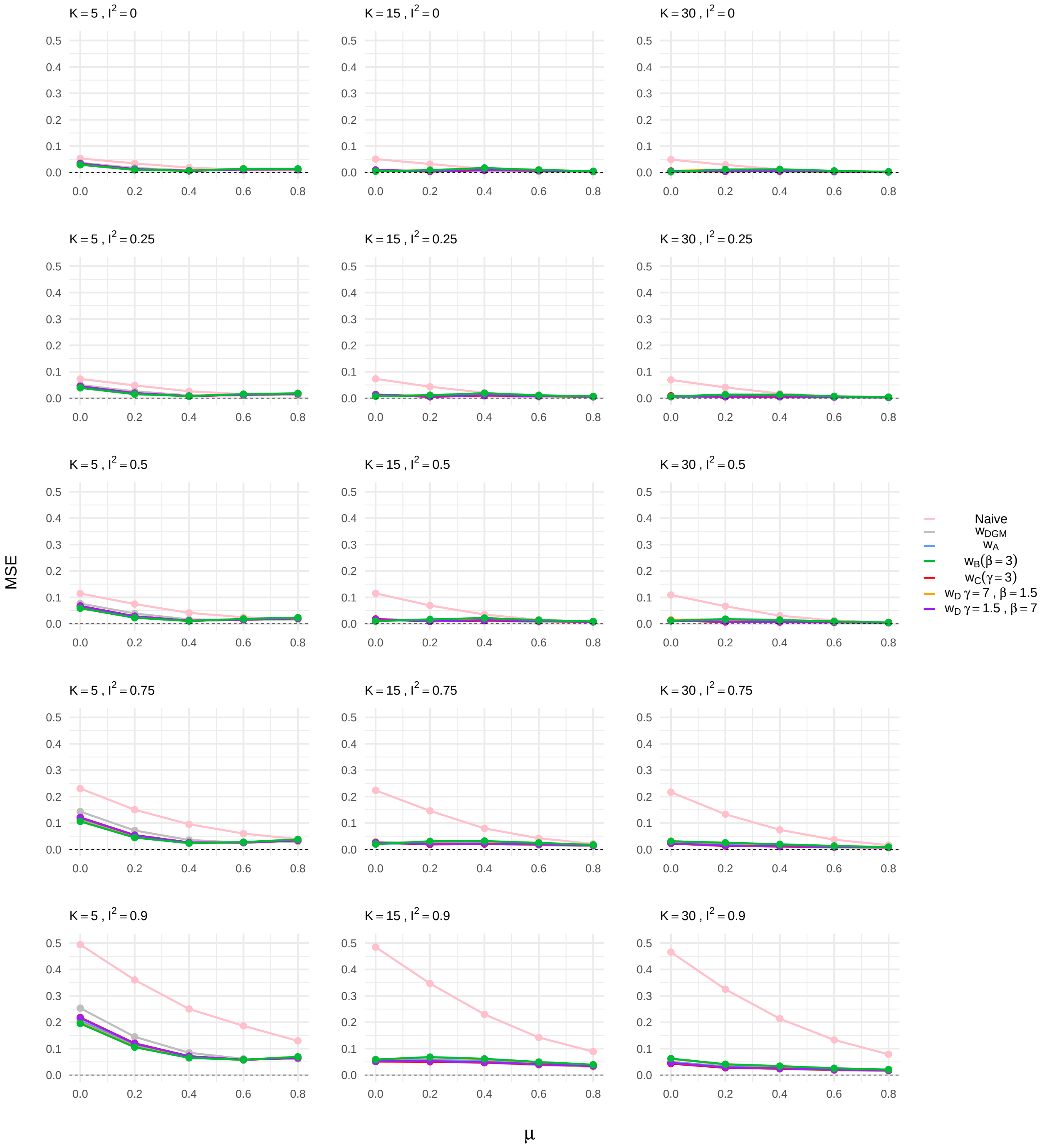

Figure 3: Coverage in the estimation of the treatment effect μ for ORB simulated according to DGM function with $\gamma = 0.5$, using different estimation methods, i.e., naive or ORB-adjusted according to the various selection functions indicated in the legend. The coverage is shown for varying meta-analysis study sizes, heterogeneity levels, and increasing true treatment effect on the x-axis of each plot shown.

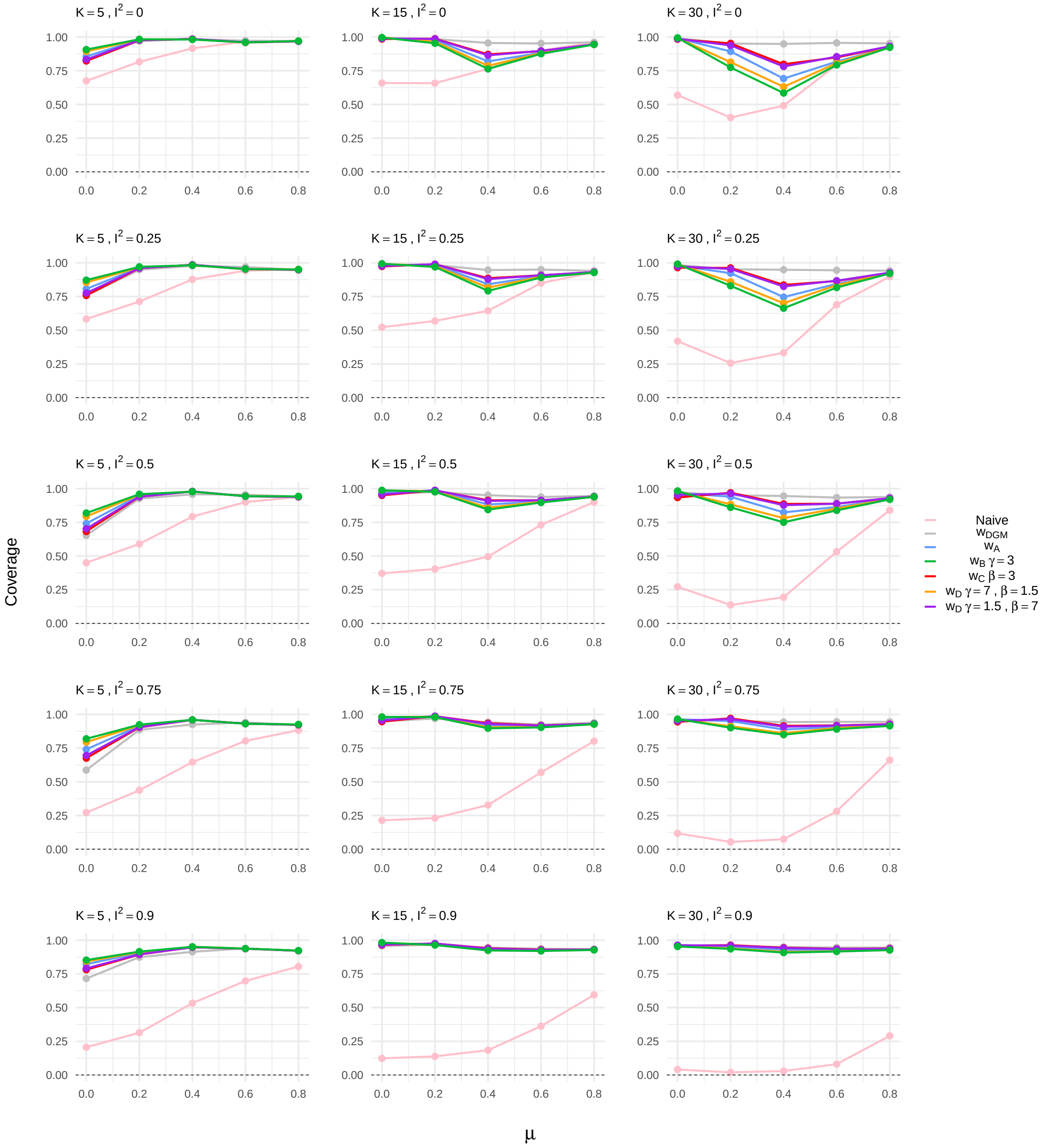

Figure 4: Power in the estimation of the treatment effect μ for ORB simulated according to DGM function with $\gamma = 1.5$, using different estimation methods, i.e., naive or ORB-adjusted according to the various selection functions indicated in the legend. The power is shown for varying meta-analysis study sizes, heterogeneity levels, and increasing true treatment effect on the x-axis of each plot shown.

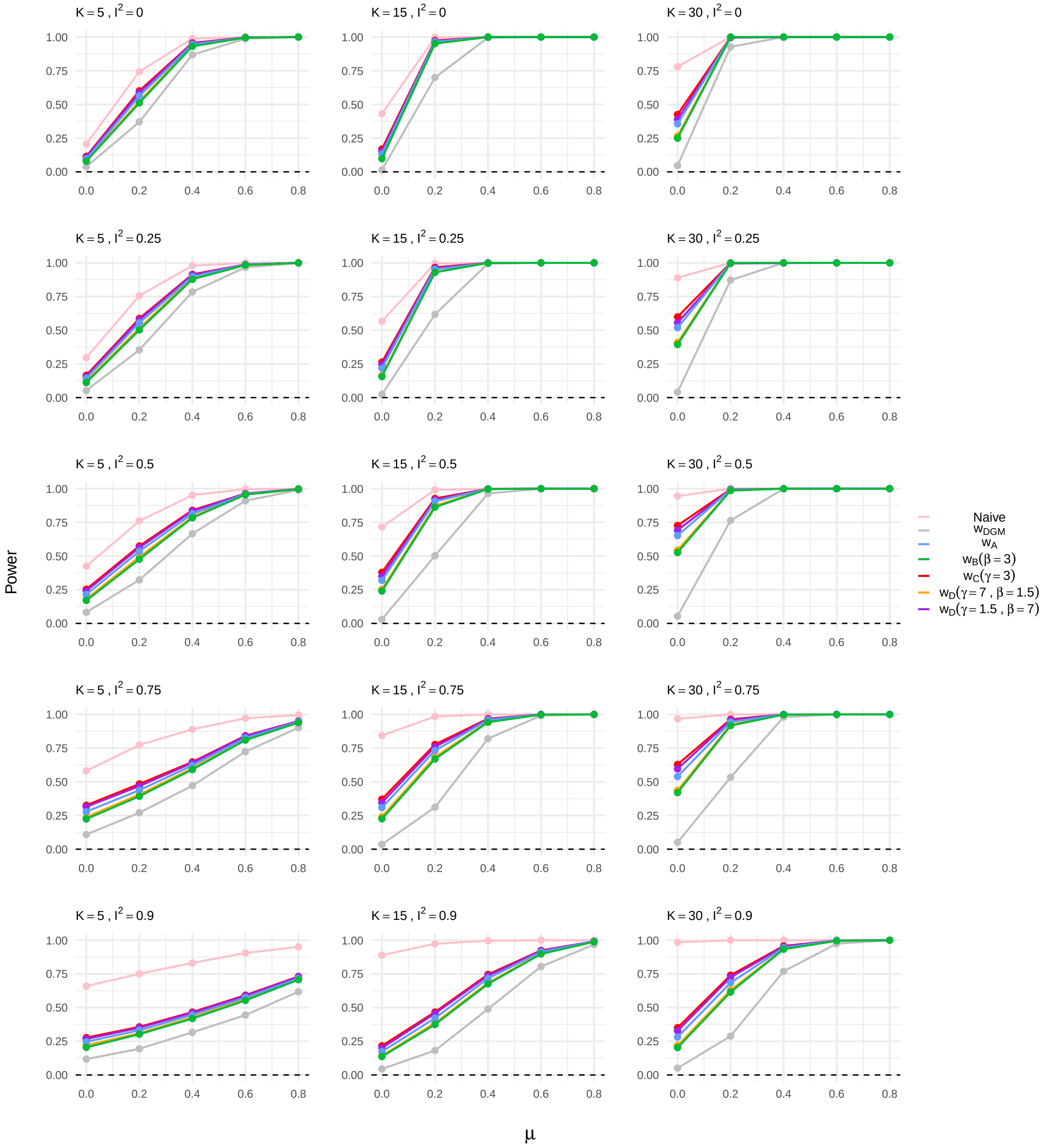

Figure 5: Power in the estimation of the treatment effect μ for ORB simulated according to DGM function with $\gamma = 0.5$, using different estimation methods, i.e., naive or ORB-adjusted according to the various selection functions indicated in the legend. The power is shown for varying meta-analysis study sizes, heterogeneity levels, and increasing true treatment effect on the x-axis of each plot shown.

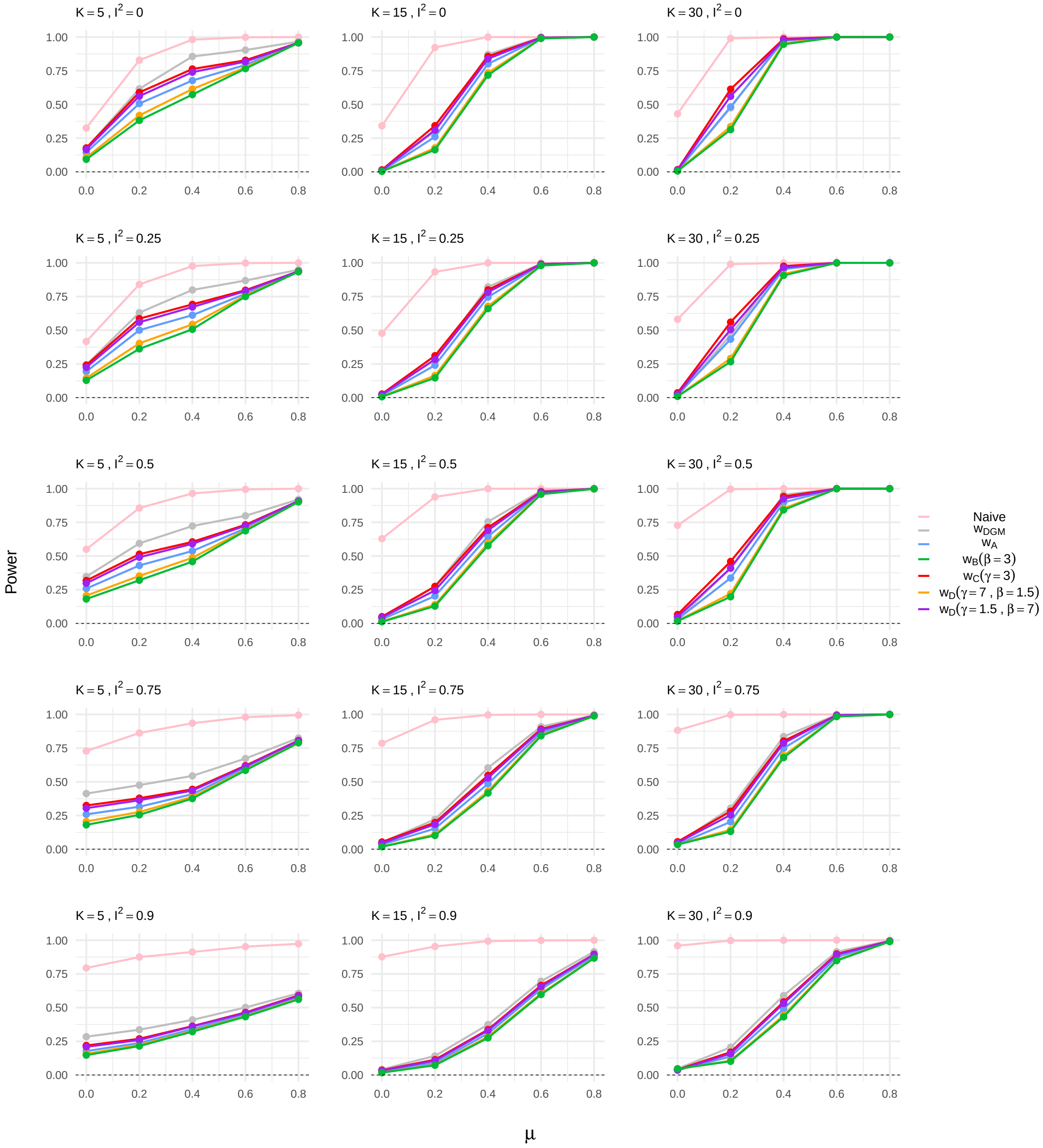

Figure 6: Empirical SE in the estimation of the treatment effect μ for ORB simulated according to DGM function with $\gamma = 1.5$, using different estimation methods, i.e., naive or ORB-adjusted according to the various selection functions indicated in the legend. The empirical SE is shown for varying meta-analysis study sizes, heterogeneity levels, and increasing true treatment effect on the x-axis of each plot shown.

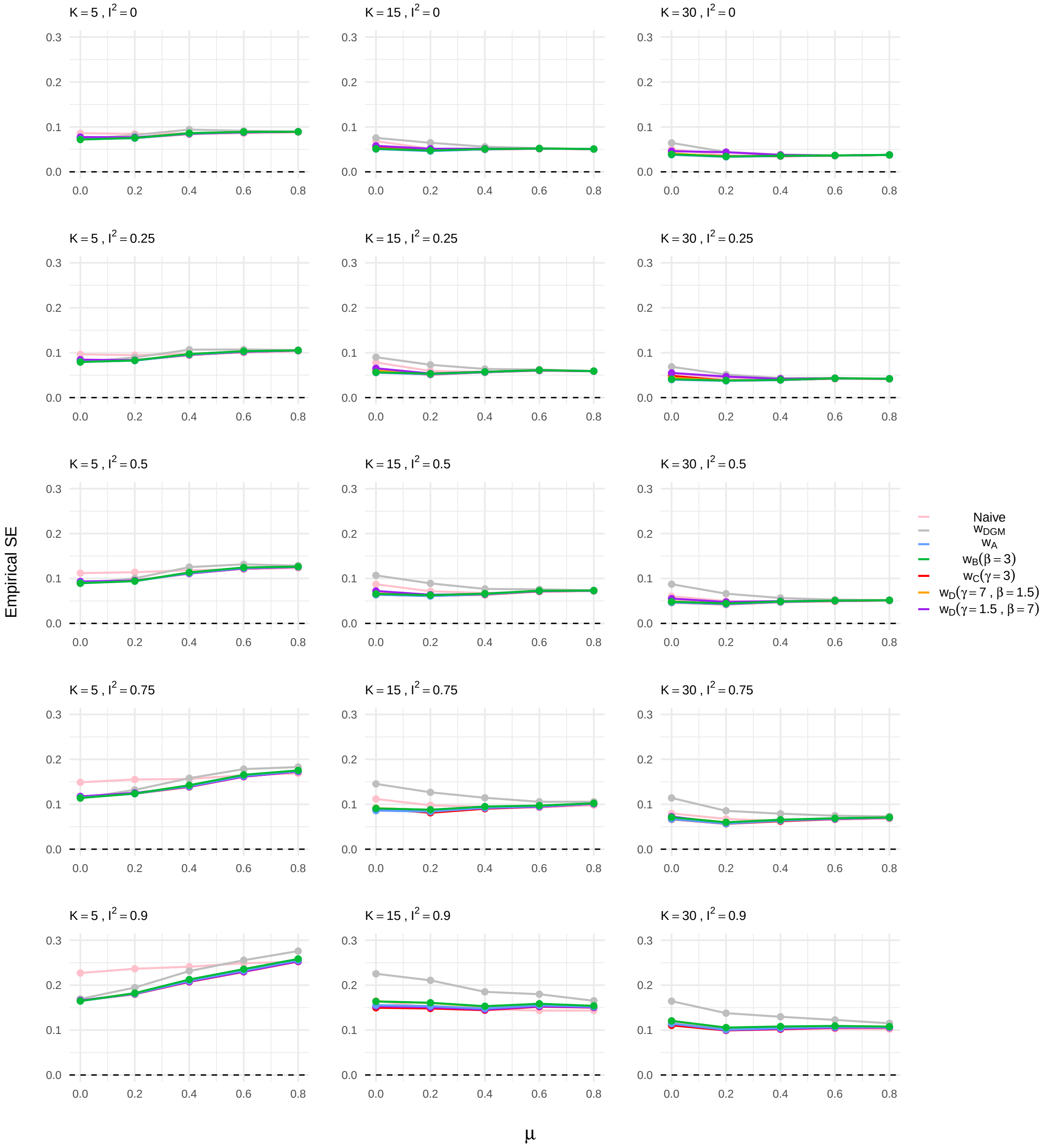

Figure 7: Empirical SE in the estimation of the treatment effect μ for ORB simulated according to DGM function with $\gamma = 0.5$, using different estimation methods, i.e., naive or ORB-adjusted according to the various selection functions indicated in the legend. The empirical SE is shown for varying meta-analysis study sizes, heterogeneity levels, and increasing true treatment effect on the x-axis of each plot shown.

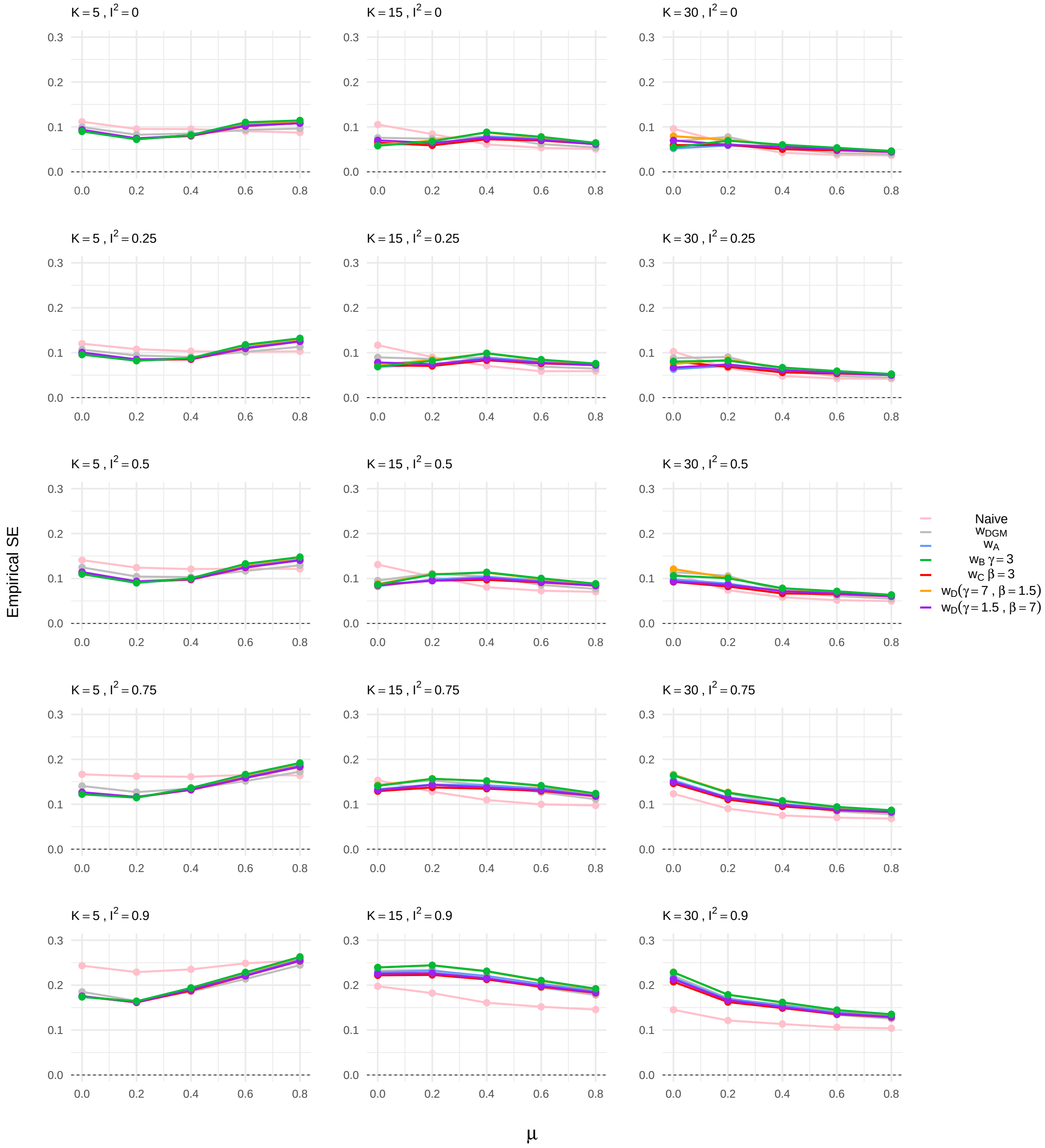

Figure 8: Bias in the estimation of the heterogeneity variance τ^2 for ORB simulated according to DGM function with $\gamma = 0.5$, using different estimation methods, i.e., naive or ORB-adjusted according to the various selection functions indicated in the legend. The bias is shown for varying meta-analysis study sizes, true treatment effect values, and increasing heterogeneity on the x-axis of each plot shown.

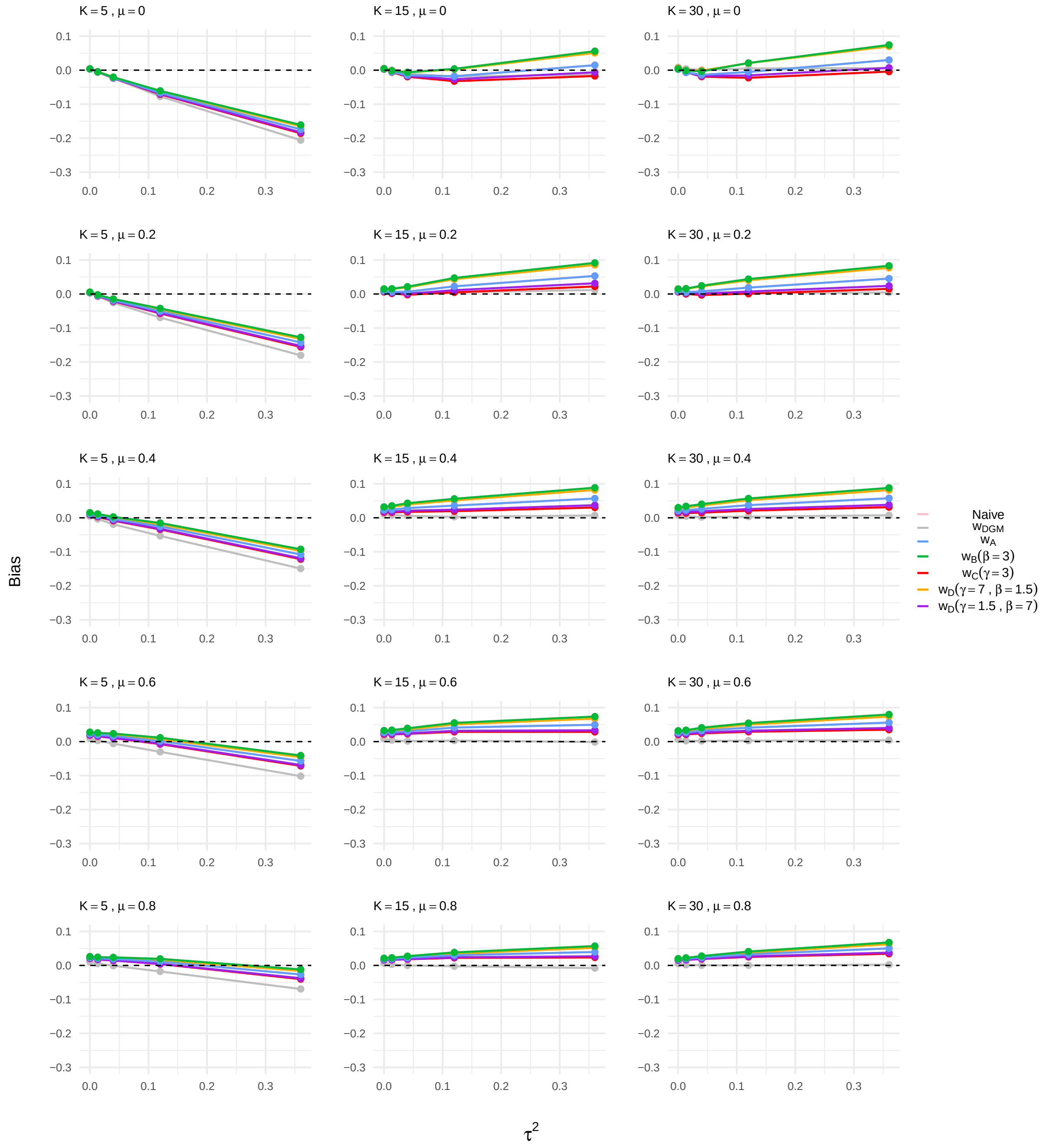

Figure 9: Bias in the estimation of the treatment effect μ under a MCAR mechanism for studies in the meta-analysis, using different estimation methods, i.e., naive or ORB-adjusted according to the various selection functions indicated in the legend, i.e., assuming a MNAR mechanism. The bias is shown for varying meta-analysis study sizes, heterogeneity levels, and increasing true treatment effect on the x-axis of each plot shown.

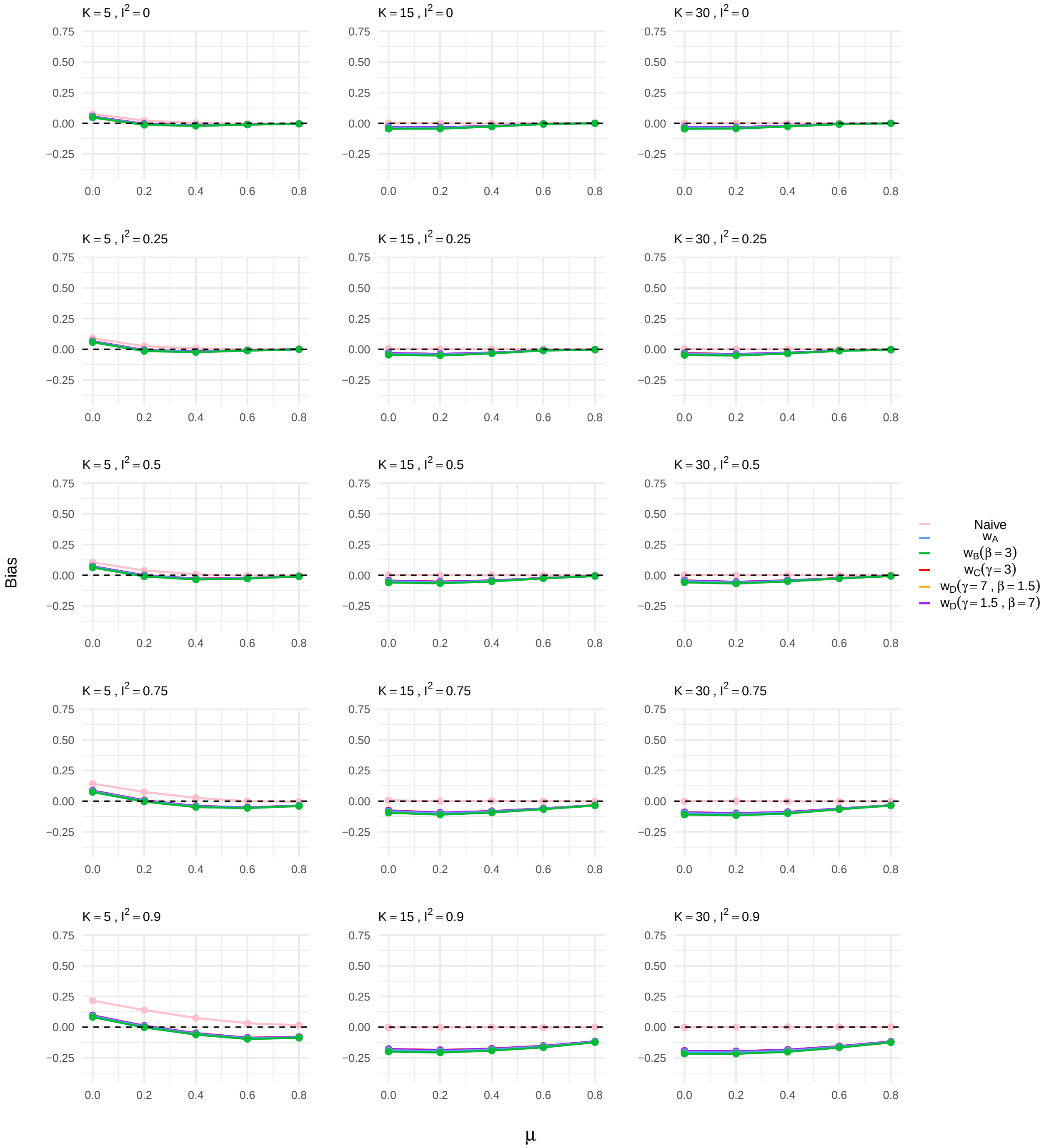

Figure 10: Bias in the estimation of the heterogeneity variance τ^2 under a MCAR mechanism for studies in the meta-analysis, using different estimation methods, i.e., naive or ORB-adjusted according to the various selection functions indicated in the legend, i.e., assuming a MNAR mechanism. The bias is shown for varying meta-analysis study sizes, true treatment effect values, and increasing heterogeneity on the x-axis of each plot shown.

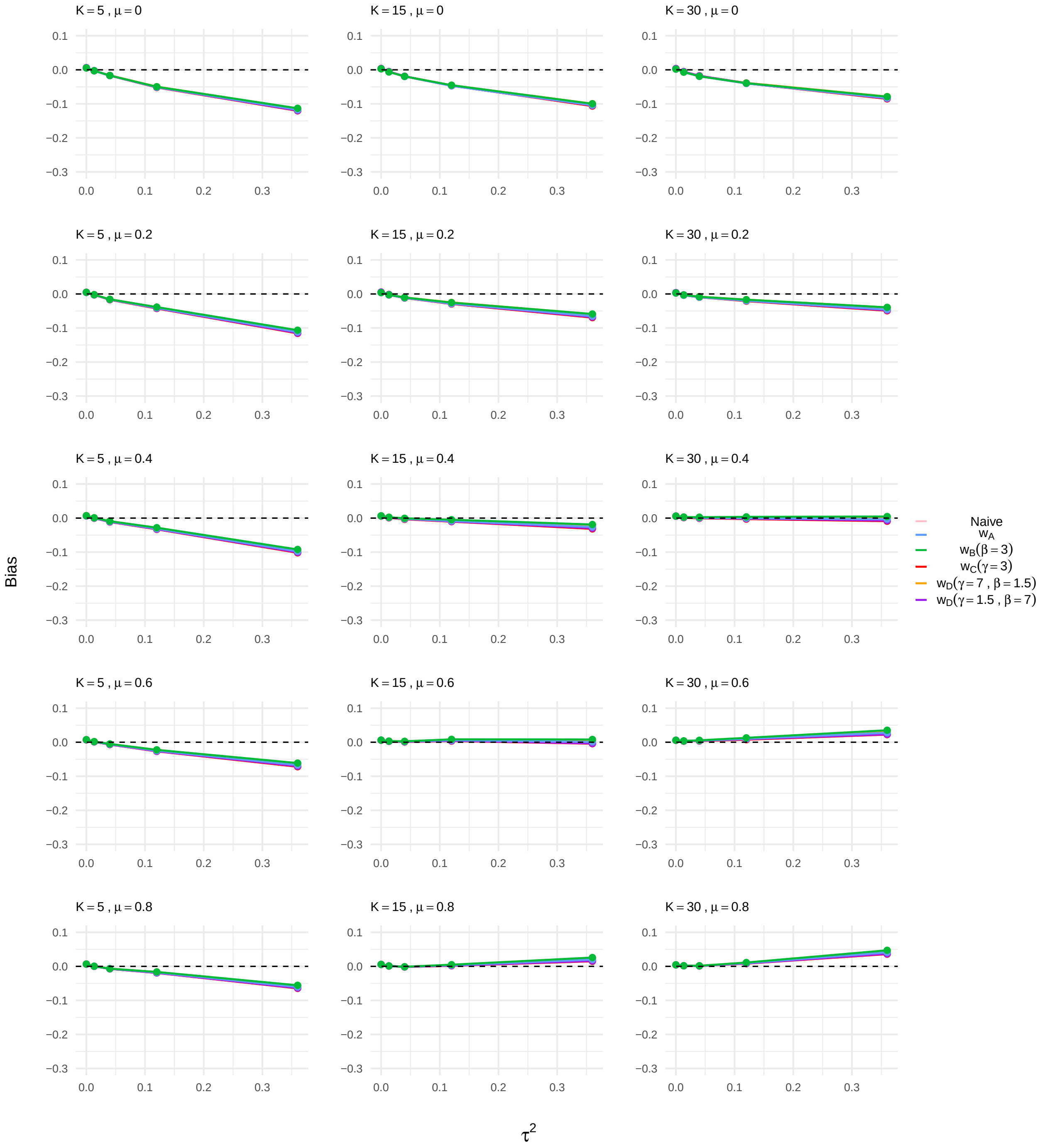

Supplement: Supplementary file 1 — Data S1: Supporting Information. [file SIM-44-0-s001.pdf]
